# Egress of non-enveloped enteric RNA viruses

**DOI:** 10.1099/jgv.0.001557

**Published:** 2021-02-09

**Authors:** Irene A. Owusu, Osbourne Quaye, Karla D. Passalacqua, Christiane E. Wobus

**Affiliations:** ^1^​ Department of Microbiology and Immunology, University of Michigan Medical School, Ann Arbor, MI 48109-5620, USA; ^2^​ West African Centre for Cell Biology of Infectious Pathogens, Department of Biochemistry, Cell and Molecular Biology, University of Ghana, Legon, Accra, Ghana; ^3^​ Henry Ford Health System, Detroit, MI 48202, USA

**Keywords:** egress, enteric virus, non-enveloped virus, RNA virus

## Abstract

A long-standing paradigm in virology was that non-enveloped viruses induce cell lysis to release progeny virions. However, emerging evidence indicates that some non-enveloped viruses exit cells without inducing cell lysis, while others engage both lytic and non-lytic egress mechanisms. Enteric viruses are transmitted via the faecal–oral route and are important causes of a wide range of human infections, both gastrointestinal and extra-intestinal. Virus cellular egress, when fully understood, may be a relevant target for antiviral therapies, which could minimize the public health impact of these infections. In this review, we outline lytic and non-lytic cell egress mechanisms of non-enveloped enteric RNA viruses belonging to five families: *Picornaviridae*, *Reoviridae*, *Caliciviridae*, *Astroviridae* and *Hepeviridae*. We discuss factors that contribute to egress mechanisms and the relevance of these mechanisms to virion stability, infectivity and transmission. Since most data were obtained in traditional two-dimensional cell cultures, we will further attempt to place them into the context of polarized cultures and *in vivo* pathogenesis. Throughout the review, we highlight numerous knowledge gaps to stimulate future research into the egress mechanisms of these highly prevalent but largely understudied viruses.

## Introduction

The presence or lack of a viral envelope is a critical factor in the viral life cycle that distinguishes the cellular entry and exit strategies of enveloped from non-enveloped viruses. The lipid membrane and viral glycoproteins present in enveloped viruses dictates membrane fusion following engagement with cellular receptors for entry, and this structure typically leads to non-lytic cell exit pathways such as budding and exocytosis, although exceptions exist. Most enteric viruses are non-enveloped, except for several enveloped enteric viruses in the family *Coronaviridae*, including transmissible gastroenteritis virus (TEGV) and porcine deltacoronavirus (PdCV). These viruses can induce cell lysis via necrosis in gastric pits and small intestines in the case of PdCV [1], while TEGV infection in intestinal epithelial cells induces mitophagy [2]. Although whether induction of necrosis or mitophagy are required for viral release is not yet known.

In contrast, non-enveloped viruses traditionally have been thought to be released lytically as a result of cell death; however, recent evidence indicates that non-enveloped RNA and DNA viruses can egress without lysing cells [3]. For instance, the non-enveloped DNA virus BK polyomavirus can be released through a non-lytic pathway while cellular anion homeostasis is maintained in infected renal proximal tubule epithelial (RPTE) cells [4]. Also, the non-enveloped RNA virus hepatitis A virus (HAV) rarely causes cytolytic infections and instead is non-lytically released as a ‘quasi-enveloped’ form [5]. The quasi-envelope is a cell-derived enclosure that surrounds non-enveloped virions, making them appear enveloped, but lacks viral glycoproteins, unlike classical viral envelopes [6]. While this term was first coined for hepatitis viruses [7], the functional characteristic (i.e. a lipid membrane devoid of viral glycoproteins) extends to other membrane-wrapped non-enveloped viruses. As detailed below, non-enveloped viruses of the same family, or even the same virus, can exit the same or different cell types in multiple ways. Therefore, no inferences on the egress mechanism from a given cell type or by a given virus can be made.

Knowledge of viral egress mechanisms is important for our understanding of pathogenesis, including viral entry and infection, incubation period, disease outcome and progression, and viral transmission within and between hosts. For instance, some non-enveloped viruses released non-lytically in membrane-enclosed vesicles establish infections more efficiently than native ‘naked’ virus particles [8]. Membrane enclosure also allows for neutralizing antibody evasion [9], as well as *en bloc* transmission of viruses. *En bloc* transmission increases the chances of a productive infection by promoting a higher infectious dose in instances where multiple viral particles are enclosed within a vesicle [10]. Hence, understanding various egress mechanisms can inform prognosis and management of viral infections and aid in the development of drugs or vaccines to block virus transmission.

**Fig. 1.**
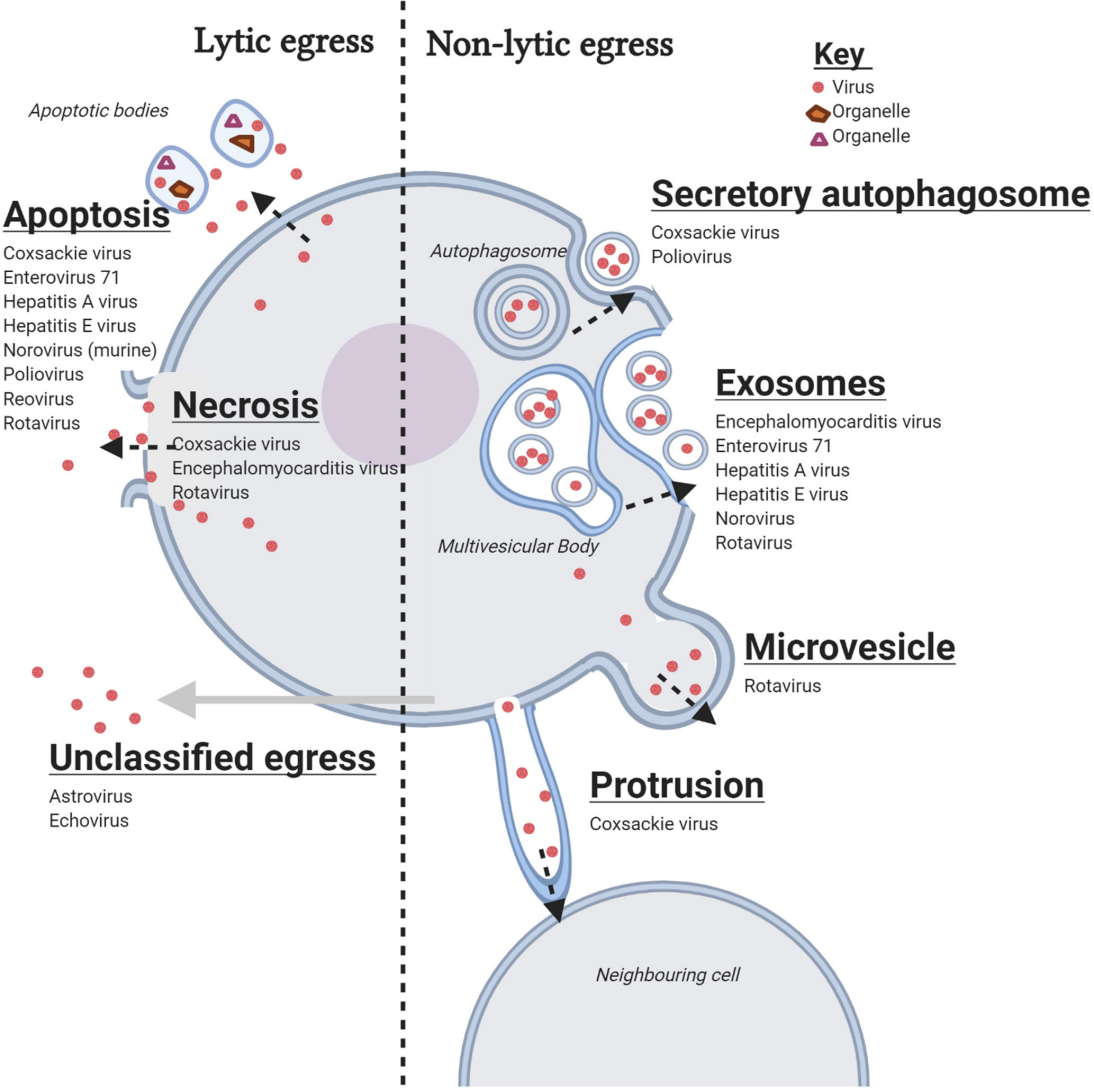
Summary of currently known egress mechanisms of non-enveloped enteric RNA viruses discussed in the text. Created with Biorender.

Non-enveloped enteric RNA viruses comprise a group of viral families that cause some common human diseases. For instance, human noroviruses in the family *Caliciviridae* are the leading cause of viral gastroenteritis globally and are responsible for about 18 % of all gastroenteritis cases [11]. Coxsackievirus, echovirus and enterovirus 71, members of the family *Picornaviridae*, are leading causes of viral meningitis and hand, foot and mouth disease, respectively [12–14]. Also, two important causes of viral hepatitis (hepatitis A and E virus) are non-enveloped enteric RNA viruses. Thus, non-enveloped enteric RNA viruses cause a significant public health burden. Understanding how they are released provides an insight into infection parameters, which has important implications for disease management and prevention. Therefore, this review will focus on the cellular egress mechanisms of faecal–orally transmitted non-enveloped RNA viruses belonging to five families; *Picornaviridae* (coxsackievirus, echovirus, encephalomyocarditis virus, enterovirus 71, hepatitis A virus/hepatovirus, poliovirus), *Reoviridae* (reovirus and rotavirus), *Caliciviridae* (norovirus), *Astroviridae* (astrovirus) and *Hepeviridae* (hepatitis E virus). Table 1, Fig. 1 outline currently known egress mechanisms for the non-enveloped enteric viruses discussed in this review.

**Table 1.** Non-enveloped enteric viruses with currently known egress mechanisms

Virus	Egress mechanism	Cell type	Reference
*Picornaviridae*			
Coxsackievirus	Caspase-dependent apoptosis	HeLa, COS-1	[25, 29, 30]
	Caspase-independent apoptosis	HeLa	[86]
	Necrosis	Caco-2	[88]
	Secretory autophagosomes (AWOL)	HeLa, MEFs, skeletal myoblasts, mouse atrial myocytes	[106, 107]
	Protrusion-mediated cell-to-cell spread	GMK	[134]
Echovirus	Strain-dependent lytic and non-lytic release	Monkey kidney cells	[141]
Encephalomyocarditis virus (EMCV)	Necrosis	HeLa, BHK-21	[89, 90]
	Exosomes	HeLa	[119]
Enterovirus 71 (EV71)	Caspase-dependent apoptosis	RD, HeLa, HMEC-1, SF268	[24, 33]
	Exosomes	Human neuroblastoma, RD, motor neurons	[8, 113]
Hepatitis A virus (HAV)	Caspase-dependent apoptosis	FRhK-4	[34]
	Exosomes	Huh 7.5	[114]
	Apical	Caco-2	[3]
	Apical and basolateral release	HepG2	[115, 149, 150]
Poliovirus	Caspase-dependent apoptosis	HeLa, U937	[26, 32, 85, 99]
	Caspase-independent apoptosis	HeLa	[85]
	Secretory autophagosomes	HeLa, human chorion cells, Huh, COS-1	[100] [19, 101–104]
	Apical release	Polarized Caco-2	[148]
	Apical and basolateral release	Polarized Vero	
*Reoviridae*			
Reovirus	Caspase-dependent apoptosis	HEK293, HeLa NIH 3T3, L-929, MEFs	[39–43, 48]
	Apical release	Human respiratory epithelial cells, HBMECs	[157, 159]
Rotavirus	Caspase-dependent apoptosis	Caco-2, HT-29, MA104	[61]
	Necrosis	MA104, L-929	[91, 92]
	Microvesicles	Human cholangiocytes, MA104, Caco-2	[10, 61]
	Apical release	IPEC-J2 Caco-2	[160, 162]
	Apical and basolateral release	MA104	
*Caliciviridae*			
Norovirus	Caspase-dependent apoptosis	Tuft cells, RAW 264.7, CrFK	[66–72]
	Exosomes	Stool (*in vivo*), RAW 264.7	[10, 121]
*Astroviridae*			
Human astrovirus	Unclassified non-lytic release	Caco-2, HEK293T, A549	[136–139]
*Herpeviridae*			
Hepatitis E virus	Caspase-dependent apoptosis	HBMECs	[77]
	Exosomes	PLC/PKF/5, A549	[122–125, 128]
	Apical release	HepG2/C3A, Caco-2 cells	[130, 154]

### Virus egress mechanisms

The way a virus exits a cell depends both on the virus itself and the cell type being infected. Thus, the outcome of infection of the same virus in different cell types is partly dictated by the underlying cellular response. In this section, we discuss three main mechanisms by which non-enveloped enteric RNA viruses are released from cells. First, we focus on the classical lytic release mechanism, where viruses exit infected cells by killing cells either via apoptosis or necrosis. Second, non-lytic release pathways, by which non-enveloped enteric RNA viruses are released without activating cell death mechanisms, are discussed. Finally, we highlight non-lytic release of viruses via cell-to-cell protrusions and discuss the directional release of viruses from polarized cells.

### Lytic virus release

Cell death can occur by multiple mechanisms, including apoptosis, necrosis and autophagy [15]. Apoptosis is an inherent programmed cell death mechanism in cells of multicellular organisms that is mediated by the formation of apoptotic bodies, which minimizes exposure of cellular contents to the extracellular space, thereby reducing the risk of inflammation [16]. Initiation of apoptosis occurs via an intrinsic (mitochondrial) or extrinsic (death receptor) pathway, and these converge in the activation of executioner caspases 3, 6, or 7. When proapoptotic molecules are released from the mitochondria without involvement of caspases, a caspase-independent cell death ensues [17]. Since most of the research we reviewed examined the involvement of caspases in cell death, this review will categorize apoptotic cell death into caspase-dependent and caspase-independent apoptosis. In addition, necrosis is a form of uncontrolled cell death initiated by external stimuli that leads to the loss of plasma membrane integrity, release of cellular contents, and a stronger inflammatory response than apoptosis [16, 18]. While pyroptosis and necroptosis are other forms of cell death that each share some characteristics with apoptosis and necrosis, a link to enteric RNA virus release has not been demonstrated to date. Thus, we will focus solely on apoptosis and necrosis. Autophagy, which ultimately can lead to cell death, will also be discussed as a means toward vesicle generation for non-lytic virus release [19–22]. It is important to note that while viral infections can cause host cell death in a variety of ways, cell death may not be directly induced by viruses for their release but may be an indirect consequence of virus-induced cellular changes. However, for the purpose of this review, we correlate host cell death and virus egress based on the observation of reduced virus release when apoptosis or necrosis is inhibited. Much less is known about such a link *in vivo*, but we point out the known examples.

#### Caspase-dependent apoptosis

Caspase-dependent apoptosis occurs when an apoptotic stimulus such as an infectious agent, an immune reaction, or a toxin induces the expression of Bax proteins. Bax in turn activates the apoptotic cascade and mediates permeabilization of the mitochondrial membrane for release of pro-apoptotic factors, including cytochrome c. Cytochrome c then activates a cascade of caspases to induce cellular changes such as DNA fragmentation, nuclear pyknosis and chromatin condensation [23]. Members of the *Picornaviridae*, *Reoviridae*, *Caliciviridae* and *Hepeviridae* induce caspase-dependent apoptosis in specific cell types, resulting in the release of virions into the cell culture supernatant.

Specifically, members of the family *Picornaviridae*, including poliovirus, coxsackievirus, enterovirus 71 (EV71) and hepatitis A virus (HAV), induce caspase-dependent apoptosis via activity of the viral non-structural protein 2B, resulting in cell lysis of transformed epithelial cell lines and virion release into the supernatant. EV71 2B localizes to the mitochondrial membrane to interact with and activate Bax [24], while coxsackievirus B3 (CVB3) 2B disrupts calcium ion homeostasis to create lesions in the cellular membrane, leading to the apoptotic release of virus progeny [25]. In addition, poliovirus, CVB3 and EV71 can also use their 3C proteases to induce caspase-dependent apoptosis by unknown mechanisms [26–30]. In the case of duck hepatitis A virus, the structural protein VP3 induces the intrinsic pathway of apoptosis in duck embryo fibroblasts upon transfection [31]. Whether the same proteins also mediate apoptotic release of poliovirus and EV71 from human macrophage (U937) and endothelial (HMEC-1) cells [32, 33], or HAV from foetal rhesus kidney-4 (FRhK-4) cells [34], remains unknown. *In vivo,* apoptosis contributes to pathogenesis because it is associated with poliovirus replication and damage to the central nervous system during paralytic poliomyelitis in infected mice [35] and humans [36, 37]. However, it is unclear whether apoptosis is important for virus release *in vivo*. In summary, egress of picornaviruses via caspase-dependent apoptosis is at least in part mediated by the 2B viroporin and/or 3C viral protease. The role of 2B protein in mediating caspase-dependent apoptosis in infected cells is shared by viroporins of other RNA viruses such as Sindbis virus (6K), mouse hepatitis virus (E), influenza virus (M2) and hepatis C virus (P7 and NS4A) [38].

Caspase-dependent apoptosis and virus egress occur in *Reoviridae* (reovirus and rotavirus) infection via different mechanisms. Reovirus infection can induce caspase-mediated apoptosis in transformed and primary cells *in vitro* [39–48], depending on the virus strain. These virus strain-specific differences in the ability to induce apoptosis are linked to the S1 and M2 gene segments, encoding the viral cell attachment protein, σ1, and a small non-structural protein, σ1s, as well as the major outer capsid protein, *μ*1, respectively [49].

*In vivo,* reovirus-induced apoptosis in the heart and nervous system is mediated by σ1s [50]. This viral protein localizes to the nucleus of cells to disrupt the nuclear landscape, including the A-type nuclear lamin-network (La A/C) [51]. Disruption of La A/C in heart muscle cells weakens the nucleus, which, coupled with the constant mechanical stress of a beating heart, drives cells towards apoptosis [52]. In the nervous system, σ1s enhances cell cycle arrest at the G2/M phase to inhibit proliferation of reovirus-infected neurons, resulting in apoptosis [53–55]. Whether apoptosis in these organs leads to virus release remains unknown. This contrasts with the intestine, where apoptosis is linked to virus release. Specifically, the non-apoptotic strain of reovirus T1L persists in the intestine, while the apoptotic strain T3D-RV is cleared more rapidly due to the sloughing off of infected apoptotic cells [56]. Induction of apoptosis in the intestine is mediated by the M1 and M2 viral genes [56]. Thus, for reoviruses, host (i.e. organ type) and viral (e.g. σ1s, *μ*1 and *μ*2 proteins) factors mediate virus release.

During rotavirus infection *in vitro*, induction of caspase-dependent apoptosis and virus release into the supernatant is cell type- and cell differentiation-dependent (Table 1). Infection of transformed human colon cancer cell lines (Caco-2, HT-29) and MA104 cells (African green monkey kidney) with simian rhesus rotavirus RRV and porcine rotavirus CRW-8 strains leads to typical apoptotic features, such as DNA fragmentation, chromatin condensation, release of apoptotic bodies, disruption of mitochondrial membrane potential, release of cytochrome c and caspase activation [57–61]. However, in Caco-2 cells, apoptosis only occurs in fully differentiated cells [57, 59]. Induction of apoptosis is mediated by the NSP4 protein of rotavirus, which translocates to and disrupts the mitochondrial membrane, causing release of cytochrome c and triggering caspase activation [62]. NSP4 also triggers dynamic Ca^2+^ signalling [63], which when prolonged results in cell lysis. *In vivo*, rotavirus infection causes apoptosis of infected enterocytes, which underlies the pathobiology of disease, including villus atrophy [64]. The loss of dying cells from the intestinal epithelium in turn may mediate viral dissemination to new hosts. In summary, induction of caspase-dependent apoptosis by members of the family *Reoviridae* is mediated either by structural proteins (i.e. reovirus *μ*1 and *μ*2), or non-structural proteins (i.e. rotavirus NSP4, reovirus σ1s) *in vitro* and *in vivo*, highlighting the importance of this function during infection.

In the case of caliciviruses, murine norovirus (MNV) infection of murine macrophages and dendritic cells causes cytopathic effect (CPE) [65], suggesting that these viruses induce cell death to enable egress. The visible CPE and loss of cell viability seen with propidium iodide staining was also observed with MNV infection in an immature B cell line (WEHI-231) [66]. Optimal virus release of MNV from infected murine macrophages (RAW 264.7 cells) is due to caspase-mediated apoptosis, which is tightly regulated by the virulence factor 1 (VF1) and downregulation of the anti-apoptotic protein survivin by a yet unidentified viral non-structural protein [67–70]. Similarly, feline calicivirus (FCV) downregulates survivin and XIAP (X-linked inhibitor of apoptosis protein) to induce caspase-dependent apoptosis in feline kidney (CrFK) cells [71]. Downregulation of survivin is mediated by the FCV leader capsid (LC) protein [71]. *In vivo*, the persistent MNV strain CR6, but not the acute MNV-1 strain, induces apoptotic cell death of tuft cells for sustained shedding and transmission of virus in faeces [72, 73], representing an example that links apoptosis to virus release from the host.

For human norovirus (HNoV), the strongest evidence that infection induces caspase-dependent apoptosis comes from analysis of intestinal biopsies from HNoV-infected individuals that showed evidence of DNA fragmentation and caspase activation in duodenal epithelial cells [74]. However, whether apoptosis is required for virion release is unknown. *In vitro*, HNoV-infected human intestinal enteroids show limited cell rounding and cell death [75], and no cell death is detected in mature B-cell cultures infected with HNoV [66]. Thus, while HNoV can cause cell death *in vivo*, permissive cells in culture do not exhibit widespread CPE, suggesting an alternative egress pathway under these conditions. Collectively, studies to date indicate that *Caliciviridae* induce lytic release via caspase-dependent apoptosis following downregulation of survivin by structural (FCV) or non-structural proteins (MNV). Future studies are needed to determine the molecular mechanism and viral mediators of HNoV-induced apoptosis.

Hepatitis E virus (HEV) causes cell injury by inducing an intrinsic caspase-mediated apoptotic pathway in the liver [76] and brain *in vivo* and in primary human brain microvasculature endothelial cells *in vitro* [77]. Tissue sections from HEV-infected Mongolian gerbils showed increased TUNEL staining and increased expression of caspase-9, caspase-3 and the pro-apoptotic protein Bax, a finding also made *in vitro* [77]. Apoptotic cell death and associated tissue injury in the brain and liver may account for the neurological disorders and hepatitis associated with HEV infection [78, 79]. However, whether cellular apoptosis is required for HEV release and transmission or the viral proteins triggering apoptosis remain to be explored.

In summary, caspase-dependent apoptosis is induced by many non-enveloped RNA viruses, resulting in virion release into the cell culture supernatant. *In vivo*, apoptosis of virus-infected intestinal epithelial cells is an important characteristic of pathogenesis, as it ensures virion release and subsequent transmission to new hosts. Initiation of this process typically occurs via the intrinsic pathway, given the obligate intracellular nature of viruses, and is mediated by specific non-structural or structural viral proteins, depending on the virus species.

#### Caspase-independent apoptosis

Caspase-independent apoptosis occurs when the mitochondrial membrane potential is disrupted through calcium ion dysregulation or reactive oxygen species, leading to translocation of apoptotic proteins such as apoptosis-inducing factor (AIF) and endonuclease G from mitochondria to the nucleus. This process in turn induces chromatin condensation and/or large-scale DNA fragmentation with no caspase activation [23, 80, 81]. Generally, the kinetics of apoptosis are slower with caspase-independence, and cells are likely to persist longer, since they do not expose phosphatidylserine, which is a detection signal for destruction of apoptotic cells [82]. RNA viruses known to induce caspase-independent apoptosis include porcine epidemic diarrhoea virus (PEDV) and hepatitis C virus [83, 84]. Among non-enveloped enteric RNA viruses, the family *Picornaviridae* members poliovirus and coxsackievirus have been shown to induce caspase-independent apoptosis in specific cells for viral release and spread.

Poliovirus and CVB3 also induce apoptosis in HeLa cells without caspase activation [85, 86]. Cells infected with these viruses exhibit signs of apoptosis such as DNA fragmentation, chromatin condensation, and nuclear deformation during caspase inhibition, indicating that apoptotic cell death does not solely depend on caspase activation in HeLa cells. Unlike in caspase-dependent apoptosis, where the 3C and 2B viral proteins are involved in inducing the process, no viral protein(s) has been implicated in caspase-independent apoptosis in picornaviruses. Thus, although these viruses can induce both caspase-dependent and- independent apoptosis, the factors that determine the type of apoptotic cell death induced during an infection and the regulation of these pathways are unknown.

#### Necrosis

Necrosis is a form of cell death that occurs with the loss of plasma membrane integrity that is preceded by swelling of cellular organelles, ultimately leading to the release of cellular contents to the extracellular space [16, 18]. The exposure of cytoplasmic content during necrosis provokes an inflammatory response around the dying cell that catalyses pathological processes [87]. Necrotic cells generally exhibit vacuolation of the cytoplasm, breakdown of the plasma membrane and changes in nuclear morphology, but not DNA fragmentation and chromatin condensation, as seen in apoptosis [15]. Calpain and cathepsin are major proteases involved in necrotic cell death [18]. Viruses in the families *Picornaviridae* and *Reoviridae* induce necrosis for viral release and spread.

The *Picornaviridae* members CVB1 and encephalomyocarditis virus (EMCV) exit Caco-2, HeLa, or baby hamster kidney (BHK-21) cells through necrotic cell death [88–90]. No signs of apoptosis, such as externalization of phosphatidylserine, pronounced chromatin condensation, DNA fragmentation, caspase cleavage and cytoplasmic blebbing, are observed in infected cells, yet damage to cell membranes leading to cell death occurs, suggestive of necrosis. CVB1 induces necrotic death in Caco-2 cells by hijacking the Ca^2+^ signalling pathway [88], while during EMCV infection of HeLa and BHK-21, the leader (L) and 2A proteins induce necrosis and suppress apoptosis for optimal viral yield [89, 90]. The mechanism of necrosis induction and the relative importance of apoptosis versus necrosis during pathogenesis *in vivo* remain unknown.

In the family *Reoviridae*, two strains of rotavirus, the simian strain SA-11 and the porcine strain 1154, lyse MA104 cells mainly via necrosis [91], whereas the highly cytolytic T3D reovirus strain kills L-929 cells via necrosis and apoptosis [92]. Infected cells exhibit signs such as damage to the cell membrane, which leads to cell content leakage and nuclear fragmentation without DNA cleavage, indicating that CPE is due to necrosis but not apoptosis. The kinase activity of receptor-interacting protein 1 (RIP1) is required for necrosis during reovirus infection [92], while the cellular factors necessary for rotavirus-induced necrosis are unknown. Future studies are needed to identify viral and host determinants, which pathways are engaged, and what mechanisms control them.

Collectively, the evidence so far suggests that most non-enveloped enteric RNA viruses induce cell death by caspase-dependent apoptosis, while the induction of caspase-independent apoptosis and necrosis are reported less often (Table 1). The involvement of the picornavirus 2B and 3C proteins as inducers of apoptosis provides critical knowledge for our mechanistic understanding. It is important, however, to note that much of this research has been performed in transformed cell lines in culture and using cell culture-adapted virus strains. Thus, validation of findings with clinical isolates and in physiologically relevant non-transformed cell culture models and/or *in vivo* will be important in the future. In addition, much remains unknown about the identity and mechanisms by which viral proteins of different viral families induce cell death either *in vitro* or *in vivo*. Furthermore, for those viruses that can induce cell death by multiple mechanisms, determining the factors (viral and/or host) that drive engagement of one pathway over another and the consequences on viral pathogenesis will add to our fundamental understanding of virology.

### Non-lytic release

In the absence of cell death, egress of non-enveloped enteric RNA viruses into the extracellular space can also occur through membranous enclosure of progeny virus and non-lytic release via vesicles. Extracellular vesicles (EVs) are released by most eukaryotic and prokaryotic cells to modulate cell behaviour [93, 94], and they originate from different cellular pathways. Based on size and how they are derived, EVs are classified into microvesicles, secretory autophagosomes and exosomes [95]. Here, we describe the non-lytic release of non-enveloped enteric RNA viruses in all three EV types (Fig. 1). Also discussed is a fourth category (*others*) that describes a non-lytic viral release mechanism without the involvement of EVs.

#### Microvesicles

Microvesicles (MVs) are EVs formed from direct budding and fission of the plasma membrane into the extracellular space for release of vesicles containing cytoplasmic contents [96]. Compared to secretory autophagosomes and exosomes, MVs are the largest EVs, with diameters ranging from 200 nm to several microns. Like other EVs, MVs have inverted phosphatidylserine in the membrane [96] that can be detected by annexin V staining. Specific universal markers for MVs have not been identified, but MVs contain markers that depend on the parent cells or cellular processes that led to their release [96]. For virus release, virions are enclosed within the ‘bulged out’ cell membrane alongside other cytoplasmic contents, and the vesicles then bud off into the extracellular space without causing cell lysis.

Rotaviruses are released within MVs both *in vivo* (found in faeces) and *in vitro* [10]. Extracellular media from rotavirus-infected H69 human cholangiocytes and MA104 cells contain multiple rotavirus particles enclosed in large CD98-positive vesicles (>200 nm) that sediment at 10 000 ***g*** and have membrane-exposed phosphatidylserine [10]. Electron microscopy of phosphatidylserine-positive vesicles pulled down from stools of rotavirus-infected mouse pups and gnotobiotic piglets also revealed multiple rotavirus particles enclosed in vesicles, and fluorescence microscopy has shown that the virus-containing EVs are microvesicles of >500 nm in diameter. One intriguing observation was that, at least in MA104 cells, rotavirus particles are first released non-lytically, while lytic release occurred later in infection [10]. This suggests that viruses may have the ability to switch between different release mechanisms. However, how such a switch might be regulated, the viral and host factors involved, and the molecular mechanisms employed remain to be determined in future studies.

#### Secretory autophagosomes

Secretory autophagosomes are formed when double-membraned autophagosomes fuse with the plasma membrane, resulting in release of single-membraned vesicles of about 300–500 nm in diameter [97, 98]. Molecular markers of secretory autophagosomes include LC3 (an autophagosome membrane protein) and inverted phosphatidylserine [98]. In particular, picornaviruses are known for their subversion of autophagy, for example they prevent maturation of autophagosomes to escape degradation and can use this pathway for non-lytic release [20].

Non-lytic release of poliovirus was first suggested in the 1950s when researchers observed ‘bubbling’ and vacuolation in infected fibroblasts from adult human tonsils [99]. A decade later, non-lytic poliovirus release by extracellular vesicles likely derived from the endoplasmic reticulum, the origin of autophagosome membranes, was shown [100]. This suggests that the ‘vacuoles’ are likely secretory autophagosomes. The autophagic origin of double-membrane structures and their association with poliovirus proteins 2C and 3B was demonstrated many years later [101]. However, the presence of poliovirus particles within these vesicles was not demonstrated directly until half a century after the original discovery [19, 21, 102–104]. Detection of LC3 and lysosomal-associated membrane protein 1 (LAMP-1) on poliovirus capsid-containing vesicles confirmed the autophagosomal origin of these vesicles. Efficient poliovirus maturation into infectious virions relies on the formation of amphisomes (i.e. the fusion of autophagosome and endosome) [98] and subsequent acidification, since inhibiting acidification reduces the cleavage of VP0 capsid protein into VP2 and VP4 for maturation in cell lysates [105]. Hence, autophagic organelles remain mature and functional during infection. This non-lytic release of cell contents and virus from poliovirus-infected cells by vesicles of autophagic origin has been termed autophagosome-mediated exit without lysis (AWOL) [21]. CVB3 can also be released from cells in EVs through an AWOL-like pathway in multiple cell types *in vitro* [106, 107]. By modulating the autophagy pathway via inducing formation of autophagosome-like vesicles and inhibiting vesicle maturation and its degradative function, CVB3 enhances viral replication [108, 109]. Non-lytic release of picornaviruses using AWOL and generation of vesicles enclosing multiple virions enhances virus infection by increasing efficient cell-to-cell transmission via membrane fusion and via increased multiplicity of infection [102]. This higher local multiplicity of infection may also increase infectivity *in vivo*, as was shown for rotavirus [10]. However, future studies are needed to test the importance of vesicles containing multiple picornaviruses during viral pathogenesis.

#### Exosomes

Exosomes are the smallest type of EV (50–200 nm in diameter) and are formed from the fusion of the plasma membrane with specialized endosomes known as multivesicular bodies (MVBs) [110]. Exosomes are identified molecularly by a number of membrane proteins and lipids that are extensively reviewed elsewhere [111, 112]. Common molecular markers used to define exosomes are CD63 and CD81 [112]. This section discusses how the picornaviruses, rotavirus, norovirus and hepatitis E virus use exosomes to exit cells without lysis.

Picornaviruses HAV, EV71 and EMCV egress cells without lysis via the exosomal pathway from multiple cell types *in vitro* (Table 1) [8, 113, 114] In HAV, the exosome enclosure is termed a quasi*-*envelope, and the associated virus is referred to as eHAV [114]. The exosomal origin of the envelope was confirmed by he expression of markers, including CD63, flotillin-1, LC3B and TAPA1, and the finding that knockdown of proteins that contribute to exosome biogenesis (ALIX and VPS4B) inhibits the release of HAV [6, 114]. *In vivo*, the quasi-envelope shields virions from antibody recognition in blood and promotes effective spread within the host [115], while naked particles are shed into the environment and transmitted to new hosts. Important differences between these two forms of particles are also observed during entry [116]. Uptake of both eHAV and nHAV into cells is dependent on clathrin, dynamin and integrin β1. However, genome release from nHAV particles occurs earlier from late endosomes relative to eHAV, which release their genome from lysosomes following degradation of the quasi-envelope, suggesting a faster replication cycle for nHAV particles. Thus, the two particle types are optimized for their different roles during spread within and between hosts, but whether differential kinetics are important at different stages during pathogenesis remains unknown.

Similarly, infection of cells with exosome-associated EV71 is more efficient than infection with free virus, as shown by increased viral RNA and VP1 yields [8]. In addition to this direct effect, EV71 can also promote its infection indirectly by inducing the exosome release of a microRNA (miR-30a) from oral epithelial cells to target MyD88 in macrophages to inhibit type I interferon production [117]. However, whether miR-30a-positive exosomes contain EV71 particles is currently unknown. *In vivo*, exosome-enclosed EV71 has been identified in plasma from EV71-infected patients with viral encephalitis, while naked EV71 has been identified in stool samples from these same patients [118]. This pattern of exosome-enclosed virus in the blood and naked virions in the stool is similar to HAV [115], where it was proposed that bile acids and digestive enzymes in the gut destroy the exosomal membrane prior to faecal shedding and that exosome-enclosed virions in plasma may be a protective mechanism shielding the virus from neutralizing antibodies and enhancing intra-host spread.

In the case of EMCV, non-lytic release of progeny virus in EVs occurs early during infection of HeLa cells prior to loss of plasma membrane integrity and cell death [119]. Intriguingly, a variety of EVs that differed in both size and molecular composition were found to be associated with infectious EMCV. Size characterization was based on ultracentrifugation at different speeds: 10 000 ***g*** for larger EVs (i.e. MVs) and 100 000 ***g*** for smaller EVs. These can be further divided into subpopulations using side and forward scatter via flow cytometry. All EVs associated with EMCV possessed flotillin-1, CD63 and CD9, which are known markers of exosomes; but the smaller (100 000 ***g***) EVs also were positive for LC3I/II, which is a known marker of secretory autophagosomes. However, since MVs are large EVs that sediment at 10 000 ***g***, it will be important to widen the array of markers for characterizing these EVs to determine whether EMCV can also be released in MVs. Thus, the specific EV type(s) involved in the non-lytic release of EMCV in different cell types as well as the biological significance of the potential release via different EV types for viral pathogenesis remain to be identified.

Notwithstanding, it is important to note that EV enclosure of EMCV significantly protects virus from antibody neutralization, allowing for efficient virus spread and propagation [119]. Collectively, the non-lytic release of some picornaviruses via exosomes is likely an important factor in viral pathogenesis, since it confers a replication advantage and presents an immune escape mechanism.

In addition to picornaviruses, there is also increasing evidence that noroviruses exit cells using a non-lytic mechanism. Recently, shedding of exosome-enclosed HNoV in faeces from infected patients was reported [10]. Electron microscopy revealed small (<200 nm) vesicles containing between one and five HNoV particles following pull-down of phosphatidylserine-containing vesicles from stool of infected patients [10]. These vesicles displayed exosome-specific tetraspanins CD63, CD9 and CD81, which are known markers of multivesicular body (MVB)-derived exosomes [112, 120]. Similarly, small phosphatidylserine-containing vesicles containing infectious MNV-1 were isolated from the extracellular culture media of infected murine macrophages. The vesicle membranes contained Bis(monoacylglycerol) phosphate (BMP), a lipid enriched in MVBs and MVB-derived exosomes [121]. Blocking exosome generation with GW4869, a neutral sphingomyelinase inhibitor, significantly reduced MNV-1 release into the cell supernatant, highlighting the role of exosomes during MNV-1 exit from cells. Very limited or no CPE has been observed during HNoV infection of human intestinal enteroids [75] and B cells [66], respectively, consistent with a non-lytic release mechanism. However, the viral and host factors mediating the release remain to be investigated.

Release of quasi-enveloped hepatitis E virus (eHEV) is also observed in cell culture [122–125]. The quasi-envelope contains trans-Golgi network protein 2, is derived from membranes of MVBs [122, 123] and requires the cytoplasmic ESCRT machinery [126], impling an exosomal origin of the membrane. The viral ORF3 protein is important for membrane association of the virus via interaction with Tsg101, a cellular player in the ESCRT pathway that is also involved in the budding of enveloped viruses [126]. Thus, ORF3 is necessary for optimal release of infectious virus particles [125, 127–130]. Similar to HAV, HEV particles in bile and faeces are ‘naked’, while those in blood and culture supernatants are membrane-cloaked [127, 129, 131]. These membrane-enclosed virions are able to escape neutralizing antibodies, allowing for efficient virus spread [9, 131]. Interestingly, the infectivity of eHEV *in vitro* is lower than that of nHEV, in part due to reduced cell attachment resulting from a lack of viral proteins on the surface of the quasi-envelope [9, 132]. Both nHEV and eHEV enter cells via clathrin-mediated endocytosis, but only the latter requires Rab5 and Rab7 activity. In addition, eHEV, but not nHEV, uncoating is dependent on the lysosomal membrane degradation process [132]. These data implicate the lysosome in the degradation of the quasi-envelope and point to two locations for genome release into the cytosol, i.e. the endosome for nHEV and the lysosome for eHEV. Taken together, the selective presence or absence of the HEV exosome-like ‘envelope’ is advantageous during pathogenesis. The non-lytic release of HEV via exosomes protects the virus from neutralizing antibodies, thereby allowing viral spread in the blood, while the loss of the ‘envelope’ in faeces allows for effective transmission of more infectious nHEV between hosts.

In summary, an exosomal origin of membranes surrounding virions is shared among several different virus families. The number of virus particles enclosed in these membranes differs between viruses, which may affect genome complementation ability and, ultimately, infectivity. Furthermore, while the naked and enveloped virus particles of HAV and HEV play similar roles during distinct stages in viral pathogenesis, the role of each particle type during infection with other viruses, such as the relevance of naked and enveloped norovirus particles in the stool, remains unknown.

### Other non-lytic mechanisms

In addition to EV-mediated mechanisms, some viruses are released without cell lysis in other ways. In this section, we discuss protrusion-mediated cell-to-cell spread of coxsackie virus and the undefined non-lytic egress mechanisms of human astrovirus and echovirus.

Multiple viruses have been observed to form actin-based connections between cells in culture [133]. Among them, CVB3 may exit infected green monkey kidney (GMK) cells directly into neighbouring cells through cell protrusions [134] without cell lysis. Infection by CVB3 or transfection of CVB3 RNA into cells induces a time-dependent formation of filamentous protrusions of the plasma membrane that eventually contact the plasma membrane of neighbouring cells. Viral capsid protein was present in cellular protrusions. Importantly, when fluorescent dextran was co-injected into cells with CVB3 RNA in the presence of neutralizing antibodies, viral proteins were observed not only in the microinjected cells, but also in surrounding cells over time. Thus, these cell-to-cell protrusions, also called tunnelling nanotubes, represent an alternative way for virus to spread between cells and to escape neutralizing antibody responses. Additional studies are needed to elucidate the importance of this mechanism for viral spread in the host and the cellular and viral regulators of this process.

Astroviruses require caspase cleavage for maturation of their capsids during egress [135]. Surprisingly, though, apoptotic cell death or other cytopathic effects are not observed in infected cell cultures, yet infectious particles are detectable in culture supernatants [136–139]. This highlights the non-apoptotic functions of caspases [140] and implies that virus egress is non-lytic; however, the mechanism of release remains to be discovered. Given the understudied nature of astroviruses, it is conceivable that EVs may mediate non-lytic virion release, but definitive experiments are needed to test this hypothesis.

Another virus that can be released non-lytically is echovirus, but the mechanism of this release is unknown. Some strains of echovirus do not form plaques at all, while others eventually induce plaque formation in monkey kidney cells several days after virus release can be detected [141]. On the other hand, echovirus causes apoptotic cell death of infected dendritic cells [142], highlighting the importance of cell type for viral egress.

In summary, at least one virus from each of five families of non-enveloped enteric RNA viruses has been shown to exit cells without causing lysis (Table 1). Except for astrovirus and echovirus, there is evidence for the use of EVs for egress in all other viruses discussed (Fig. 1), implying that the most common mechanism of non-lytic egress of non-enveloped enteric RNA viruses is via EVs. Most of the viruses employ one type of EV for egress, while a few, such as rotavirus and EMCV, likely exit cells via multiple EV types. More research is needed to explore egress of non-enveloped viruses via EVs, especially for viruses such as astroviruses that cause little to no CPE in permissive cells [143]. In addition, the viral and host factors that mediate vesicle formation and enclosure for their non-lytic release, ultimately determining the type of EV employed for egress, remain to be identified. Some enveloped viruses such as hepatitis C virus, Epstein–Barr virus and herpes simplex virus, can be enclosed in exosomes that express some viral proteins, which facilitate virus entry via membrane fusion and provide virus-specific cell tropism [144]. However, viral proteins on EVs formed from the viruses discussed in this review have not been observed. While the absence of viral proteins in EVs may reduce specific virus attachment and infectivity, receptor-independent uptake may be supported through this mechanism and result in replication beyond the primary target cells, thus contributing to pathogenesis.

### Polarized cells and directional viral release

Cell polarization is the asymmetric organization of cytoplasmic and membrane-associated domains for specialized cellular functions [145], such as maintaining a barrier within an epithelium. Epithelial cell polarity is characterized by the presence of apical and basolateral membrane domains separated by adherens and tight junctions, and these domains have unique protein and phospholipid compositions [146]. A unique endocytic pathway known as transcytosis and specialized sorting mechanisms occur in polarized cells for transport of molecules. The development of polarity [147], for example the asymmetrical distribution of a viral receptor or polarized sorting, is important for uptake and release of viruses. While polarized monolayers are viewed as more physiologically relevant in *in vitro* culture models compared to traditional two-dimensional cell culture, the studies to date still rely on transformed cell lines and mechanistic details into the vectorial aspects of the virus life cycle remain scarce. Below, we summarize the directional release of select non-enveloped enteric RNA viruses from polarized epithelial cells via the apical or basolateral surface (Table 1) and the implications for viral pathogenesis.

To enter the host, picornaviruses infect the apical surface of the intestinal epithelium. Within-host spread requires virus release from the basolateral surface, while apically shed virus promotes faecal shedding and transmission to new hosts. *In vitro*, poliovirus-infected polarized Caco-2 cells release viral progeny exclusively at the apical surface, while infected polarized Vero C1008 (green monkey kidney epithelial cells) cells release viral progeny from both the apical and basolateral surfaces in the absence of significant cell lysis [148]. However, virus infection can occur from both apical and basolateral surfaces for both cell types [148].

Similarly, HAVs are mostly released into the apical supernatant from polarized Caco-2 cells without inducing CPE [115]. Virus uptake is more efficient at the apical relative to the basolateral surface, most likely due to the increased abundance of viral receptors on the apical membrane [3]. Enhanced apical release might contribute to reinfection of intestinal cells for virus amplification, in addition to shedding into stool for transmission. By contrast, egress of HAV from polarized HepG2 hepatocytes after basolateral infection is more balanced, with approximately twice as much basolateral versus apical release of particles [115, 149, 150]. However, no difference in location of release was observed between eHAV and nHAV [115]. Basolateral release of HAV from hepatocytes directly releases virus into the blood and likely accounts for viraemia during HAV infection [151, 152], but also amplifies HAV infection of the liver via basolateral reinfection of hepatocytes [149]. On the other hand, apical release of HAV from hepatocytes delivers virus into bile, with eventual release into stool for virus transmission [153]. The apical release has been described to occur via lysosome-related organelles, in which virus particles are cleaved for maturation [150]. Taken together, egress of HAV from the apical surface of polarized intestinal cells and hepatocytes is important for shedding into faeces and virus transmission, while basolateral release of the virus, which mostly occurs in hepatocytes, contributes to viraemia.

Although HEV has a similar cell tropism to HAV, there are slight differences in its release and entry patterns. In polarized hepatocytes (HepG2/C3A), HEV infects the apical and basolateral surface with equal efficiencies, but infectious particle release occurs almost exclusively from the apical surface [154], consistent with shedding into bile. Apical release is also favoured in polarized Caco-2 cells [130]. Both apical- and basolateral-released HEVs are quasi-enveloped [154], although the mechanism of vesicle formation and release may be different between the two cell surfaces [155, 156]. These data indicate that most of the infectious HEV is released into bile and the intestinal lumen for spread to new hosts, while basolateral release and viraemia are less pronounced.

Reovirus infection in polarized human respiratory epithelial cells occurs from the basolateral surface, and virus particles are released via the apical surface with minimal detectable disruption of tight junctions [157]. This directional apical release of reovirus from respiratory epithelial cells may facilitate inter-host transmission via respiratory fluids. Although signs of apoptosis such as nuclear condensation and DNA fragmentation were not observed in infected human respiratory epithelial cells, a significant amount of dead cell debris was released into the mucus membrane and airway surface liquid [157], suggesting apoptotic extrusions as a possible apical egress pathway. Apoptotic extrusions help maintain the epithelial barrier while getting rid of cells ready for programmed death [158]. Efficient reovirus uptake and release of polarized human brain microvascular endothelial cells (HBMECs) occurs from the apical surface, yet few signs of apoptosis (as measured by TUNEL staining) or lysis are detected [159]. The mechanism of non-lytic release of reovirus is still unknown and requires future investigation.

The polarity of rotavirus uptake and release is dependent on the cell model. Polarized intestinal IPEC-J2 cells infected from the basolateral surface preferentially release virions apically [160]. However, Caco-2, MDCK-1 and CV-1 cells are efficiently infected both from apical and basolateral surfaces [161], with preferential apical release in Caco-2 cells and bidirectional release from polarized MA104 [162]. The preferential apical release from intestinal cells *in vivo* would result in the excretion of the virus in the faeces for inter-host transmission, while release from the basolateral surface would allow for viral spread to underlying tissues, leading to extra-intestinal infections within the host, antigenaemia, and viraemia [163–166].

In summary, the directional release of virions from polarized cells is an important determinant of the viral life cycle and pathogenesis. The data so far on poliovirus, HAV, HEV, reovirus and rotavirus suggest that polarized intestinal cells mostly release enteric non-enveloped RNA viruses from the apical surface (Table 1), which is relevant for the excretion and transmission of viruses to ensure circulation in the population. On the other hand, release of viruses from polarized extra-intestinal cells (hepatocytes and kidney cells) often occurs from the basolateral surface, which is clinically relevant for the spread of virus to other tissues, possibly leading to viraemia and disseminated infections. However, understanding the molecular mechanisms that drive directionality of virus release from polarized cells, including whether viruses specifically interact with molecules for apical or basolateral transport, remains an important question for future research.

## Conclusion

With the exception of *Astroviridae,* whose egress mechanism is predominantly (if not exclusively) without lysis, all other families of non-enveloped enteric RNA viruses discussed here (*Picornaviridae*, *Reoviridae*, *Caliciviridae* and *Hepeviridae*) use both lytic and non-lytic mechanisms for egress in cell culture (Table 1). The common lytic mechanisms employed are apoptosis and necrosis, while the non-lytic mechanisms are mostly via EVs and, in rare cases, cell membrane protrusions (Fig. 1). It is interesting to note that some picornaviruses*,* (e.g. poliovirus and CVB3) employ multiple lytic and non-lytic egress mechanisms *in vitro*. However, the relevant studies were performed in isolation using different cell clones and experimental conditions. Thus, investigating whether lytic and non-lytic egress occur during the same infection period of picornaviruses and the significance for pathogenesis, as well as how the different processes are regulated, will provide new insights into the biology of these viruses.

Although *in vitro* experimental models using non-polarized or polarized cell monolayers are important for mechanistic studies, how non-enveloped enteric RNA viruses are released *in vivo* or in more physiologically relevant *in vitro* models such as organoids is a major knowledge gap. Therefore, an important future direction will be to elucidate features of virus infection, including release mechanisms, in two-dimensional polarized organoid monolayers or in three-dimensional organoids, which incorporate cell orientation and multiple cell types, among others.

While we have pointed out knowledge gaps throughout, some overarching questions that remain are as follows. What viral and host factors determine the type of egress mechanism employed by a virus? Do the viruses activate different mechanisms simultaneously or in a spatial/temporal pattern? How different or similar are virus egress mechanisms in non-polarized monolayers compared to organoids or polarized cells? Which of these egress mechanisms are relevant *in vivo* in various tissues? We hope that highlighting these questions will stimulate future research into fundamental aspects of non-enveloped enteric virus biology.
